# Comparison of Recurrence and Complication Rates Following Laparoscopic Inguinal Hernia Repair among Preterm versus Full-Term Newborns: A Systematic Review and Meta-Analysis

**DOI:** 10.3390/children8100853

**Published:** 2021-09-26

**Authors:** Zenon Pogorelić, Sachit Anand, Zvonimir Križanac, Apoorv Singh

**Affiliations:** 1 Department of Pediatric Surgery, University Hospital of Split, 21000 Split, Croatia; zpogorelic@gmail.com; 2 Department of Surgery, School of Medicine, University of Split, 21000 Split, Croatia; 3 Department of Pediatric Surgery, Kokilaben Dhirubhai Ambani Hospital, Mumbai 400053, India; 4 Department of Surgery, University Hospital of Split, 21000 Split, Croatia; zvonimir.krizanac@gmail.com; 5 Department of Pediatric Surgery, AIIMS, New Delhi 110029, India; dr.singhapoorv@gmail.com

**Keywords:** inguinal hernia, children, preterm babies, laparoscopic hernia repair, herniotomy, complications

## Abstract

Background: Laparoscopic inguinal hernia repair (LHR) in children has been widely performed in the last decades, although it is still not sufficiently researched in preterm infants. This systematic review and meta-analysis compared the recurrence and complication rates following laparoscopic hernia repair among preterm (PT) versus full-term (FT) newborns. Methods: Scientific databases (PubMed, EMBASE, Scopus, and Web of Science databases) were systematically searched for relevant articles. The following terms were used: (laparoscopic hernia repair) AND (preterm). The inclusion criteria were all preterm newborns with a unilateral or bilateral inguinal hernia who underwent LHR. The main outcomes were the incidence of recurrence of hernia and the proportion of children developing postoperative complications in comparison with FT newborns following LHR. Results: The present meta-analysis included four comparative studies. Three studies had a retrospective study design while one was a prospective study. A total of 1702 children were included (PT *n* = 523, FT *n* = 1179). The incidence of hernia recurrence showed no significant difference between the PT versus FT groups (RR = 2.58, 95% CI 0.89–7.47, *p* = 0.08). A significantly higher incidence of complications was observed in the PT group compared to the FT group (RR = 4.05, 95% CI 2.11–7.77, *p* < 0.0001). The PT group of newborns accounted for 81% and 72% of the major and minor complications. The major complications were either non-surgical (i.e., severe respiratory distress requiring reintubation with prolonged ventilation (or high-frequency ventilation), seizures, bradycardia), or surgical (i.e., hydroceles requiring operative intervention and umbilical port-site hernia). Conclusions: LHR in PT infants is associated with similar recurrence rates as in FT infants. However, the incidence of complications is significantly higher in PT versus FT infants.

## 1. Introduction

Inguinal hernia is one of the most common pediatric conditions managed operatively. The incidence of inguinal hernia in full-term (FT) newborns ranges from 1% to 5% and rises to 30% in premature newborns (PT) [1]. The ratio of male versus female incidence is 4–10:1, with the majority of FT newborns presenting with right-sided inguinal hernia and the majority of PT newborns with right-sided and bilateral inguinal hernia [1,2]. Traditionally, an open surgical hernia repair has been the method of choice, with high success rates and low risk of complications [3]. The development of minimally invasive pediatric surgery in the last few decades had a wide acceptance for hernia repair with numerous new laparoscopic techniques [4,5]. In comparison with open surgery, the laparoscopic approach is equivalent in terms of surgical time, length of hospital stay (LOS), and recurrence rates, and allows the opportunity to explore and repair the contralateral side, preventing metachronous hernia [4]. However, there exists a learning curve for the laparoscopic repair of pediatric inguinal hernia, especially in preterm newborns. A recent study demonstrated that the complication and recurrence rates plateau only after a surgeon performs at least thirty cases of laparoscopic-assisted percutaneous internal ring suturing [6].

The incidence of incarceration in untreated hernias in newborns and young children varies between 6% and 18%, but it increases to as high as 31% in infancy (usually in the first few months of life), posing a significant risk of bowel strangulation as well as ovarian or testicular compromise [7]. Early repair of inguinal hernia in PT infants is desirable to prevent incarceration, but the pediatric surgeon is presented with a dilemma, taking into consideration the greater anesthetic aspect and the surgical operative risk of this subset of newborns compared with the FT newborns [8]. Cardiorespiratory complications following laparoscopic hernia repair are low in both FT and PT newborns, although the incidence is comparatively higher in PT newborns. PT newborns are at higher risk for the development of early postoperative complications because of pre-existing diseases and complicated past medical history [9].

Previous reports suggest no difference between laparoscopic hernia repair in FT and PT newborns regarding postoperative complications and that the preterm population repaired before 60 weeks of post-conceptional age had a lower recurrence rate compared to FT patients and PT newborns repaired later [10,11]. However, a consensus statement regarding this subject is lacking. The aim of this systematic review and meta-analysis was to compare the recurrence and complication rates following laparoscopic hernia repair among PT versus FT newborns.

## 2. Materials and Methods

### 2.1. Search Process

The present review was not registered in any prospective register or database. The search process was performed following the Preferred Reporting Items for Systematic Reviews and Meta-Analyses (PRISMA) guidelines [12]. The present review was not registered in any prospective register or database. Two authors (S.A. and A.S.) conducted a pilot literature search in the PubMed database on 26 July 2021 to confirm the absence of any systematic reviews on this topic. On the same day, two authors (S.A. and Z.P.) independently searched the PubMed, EMBASE, Scopus, and Web of Science databases (Annexure A). The search keywords included (laparoscopic hernia repair) AND (preterm). After identification of the total articles, the duplicate records were removed. Subsequently, the remaining records were screened as per the eligibility criteria (Table A1).

### 2.2. Inclusion/Exclusion Criteria

The inclusion criteria were: *Participants*—all preterm newborns with a unilateral or bilateral inguinal hernia; *Intervention*—laparoscopic inguinal hernia repair (LHR); *Comparison*—full-term newborns undergoing LHR; and *Outcomes*—the incidence of recurrence of hernia and the proportion of children developing postoperative complications, were the outcomes studied in this review. The complications were divided on the basis of the Clavien–Dindo classification [13]. Furthermore, these complications were sub-categorized into major (Grade 3 or above) and minor (Grade 1 and 2); and compared among the two patient groups.

An attempt was made to include all those studies where one of the outcomes of interest was reported. In addition, the studies depicting either the extracorporeal or intracorporeal technique of hernia repair or both were eligible for inclusion. Case series, review articles, editorials, and commentaries were excluded. The studies with unavailable full-texts were also excluded.

### 2.3. Data Extraction

Data from the relevant studies were synthesized in Microsoft Excel spreadsheets (Version 15.24) by two authors (S.A. and Z.P.) independently. This included the data on the number of newborns in each group, laterality of the hernia (unilateral or bilateral), the proportion of newborns presenting with incarceration, age at hernia repair (gestational age or corrected postnatal age), the proportion of newborns developing recurrence of the hernia, and the incidence of postoperative complications. In addition, important bibliographic information including the name of the author, year of publication, and type of the study design were also recorded. Any disagreements among the authors were resolved through consensus and discussion with the third author (Z.K.).

### 2.4. Quality Assessment

In this review, the Downs and Black scale [14] was utilized for the quality assessment of the included studies. This assessment was independently performed by two authors (S.A. and A.S.). The scale yielded total scores ranging from 0–32. On the basis of these scores, the risk of bias was defined as low (score > 23), moderate (score = 16–23), or high (score 0–15) in each study. The inter-observer agreement regarding the risk of bias scores was checked using the kappa statistics [15]. On the basis of the kappa values, the level of agreement (power of kappa) was declared as almost perfect (0.81–1.00), substantial (0.61–0.80), moderate (0.41–0.60), fair (0.21–0.40), and slight (0.00–0.20).

### 2.5. Statistical Analysis

For the purpose of analysis, the newborns were divided into two groups: preterm (PT) and full-term (FT) newborns. Data were expressed as numbers, proportions, averages (mean or median), and ranges. The meta-analysis was performed using RevMan 5.4 (Cochrane Collaboration, London, UK). The risk ratio with 95% confidence intervals (CI) was calculated for both outcomes (dichotomous variables). Subsequently, the pooled risk ratio was calculated using the Mantel–Haenszel method. The level of heterogeneity among the included studies was estimated using the I^2^ statistics. A random-effects model was adopted if the heterogeneity was substantial (I^2^ > 50%). The funnel plot was drawn for the assessment of publication bias when at least ten studies were included in the meta-analysis [16]. A *p*-value of <0.05 was considered statistically significant.

## 3. Results

### 3.1. Study Characteristics

**Figure 1:**
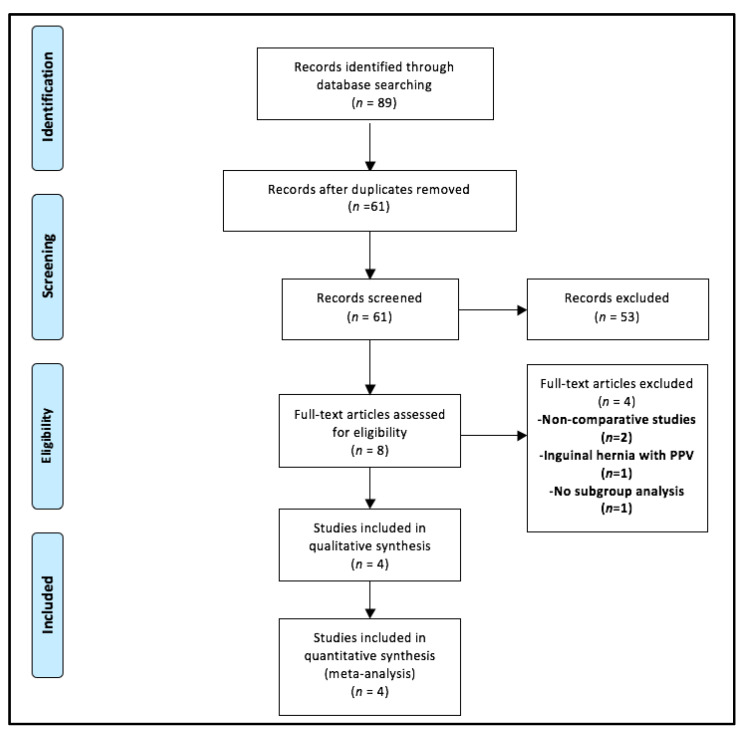
Selection of the relevant studies using the Preferred Reporting Items for Systematic Review and Meta-Analysis (PRISMA) flow diagram. Legend: PPV—patent processus vaginalis.

A total of 89 articles were identified with our search strategy (Table A1). Out of these, 28 duplicate records were removed (Figure 1). The remaining 61 abstracts were screened for eligibility. Of these, 53 were excluded and only eight articles were eligible for full-text review. Four of these were further excluded as they were non-comparative studies (*n* = 2), compared the co-occurrence of inguinal hernia and patent processus vaginalis (PPV) among FT versus PT newborns (*n* = 1), and demonstrated LHR in newborns without any subgroup analysis (*n* = 1) [1,17,18,19]. Therefore, the final meta-analysis included only four studies [9,10,11,20]. Three studies had a retrospective study design [9,10,11] while one was a prospective study [20]. A total of 1702 children were included in these studies. PT and FT groups consisted of 523 and 1179 newborns.

The baseline characteristics of the patients are depicted in Table 1. The majority of the bilateral hernias belonged to the PT group. Two studies demonstrated the incarceration rate upon presentation. Dutta et al. [20] depicted it to be significantly higher in PT newborns, however, the study by Castro et al. [10] showed no significant difference in the incarceration rates among both groups of newborns. The reporting of average age at surgery was non-uniform and is depicted in Table 1.

### 3.2. Summary of the Included Studies

#### 3.2.1. Dutta et al. (2008)

This reports a prospective study from the United States of America (USA). The authors included 187 children with 275 inguinal hernias (25 were bilateral). Of these, 49 babies (constituting 79 hernias) were preterm. Upon presentation, the incarceration rates were 18% and 4% among the PT and FT infants, respectively. A transcutaneous hernia repair was performed via the laparoscopic approach in all children. The recurrence rate was 1.5% with all recurrences noticed in the FT group. Minor complications including wound infection (1.5%), umbilical granuloma (0.7%), hydrocele (0.7%), and hematoma (2.2%) were also noticed among the study population [20].

#### 3.2.2. Burgmeier et al. (2013)

This retrospective study from Germany included a total of 336 babies with inguinal hernia. Of these, 137 and 199 babies belonged to the PT and FT groups. Intracorporeal repair using Z-type suturing was performed. The main focus of the study was to compare the cardiorespiratory complications following laparoscopic inguinal hernia repair among the two treatment groups. Of the twenty-eight PT newborns (20%) developing these complications, seven (5%) developed severe respiratory distress. In contrast, only eleven FT babies (5%) developed postoperative complications. Out of these, three (1.5%) developed severe respiratory distress. In addition, laparoscopy might have resulted in cardiac instability in a limited number of newborns. Overall, the study demonstrated a low incidence of postoperative cardiorespiratory complications among PT newborns [9].

#### 3.2.3. Castro et al. (2019)

This retrospective study was conducted by authors from Spain. A total of 156 newborns (90 PT) were included in this study. The bilateral hernia was more frequent in the PT group. Incarceration rates among the two patient groups were similar. Laparoscopic hernia repair was performed using the purse-string suture technique. The recurrence rates among the PT and FT groups were 3.2% and 0.9%, respectively. Six newborns (7%) developed minor postoperative complications in the PT group while none of the patients in the FT group developed any complications [10].

#### 3.2.4. Garcia et al. (2020)

This retrospective study from the USA included a total of 1023 patients with 1457 hernias. Of these, 247 (477 hernias) and 776 patients (980 hernias) belonged to the PT and FT groups, respectively. Bilateral hernias were more frequent among the PT group. All children underwent laparoscopic needle-assisted inguinal hernia repair. The recurrence rate was significantly higher among the FT newborns (0.82 versus 0.63; *p* = 0.025). The incidence of minor postoperative complications (including wound infection and suture granulomas) was the same among the two groups. Although the incidence of hydroceles requiring surgery was significantly (*p* < 0.01) higher among the PT newborns, the total number of complications requiring operative intervention showed no significant differences between both groups [11].

### 3.3. Methodological Quality Assessment

The Downs and Black scoring by both authors is depicted in Table 2. The average scores ranged between 16–21. All studies had a moderate risk of bias. The studies by Dutta et al. [20] and Garcia et al. [11] had the lowest and the highest scores, respectively. The inter-observer agreement was almost perfect (Kappa = 0.978; *p* < 0.001).

### 3.4. Outcome Analysis

#### 3.4.1. Meta-Analysis of Outcomes—Incidence of Recurrence of Hernia

**Figure 2:**
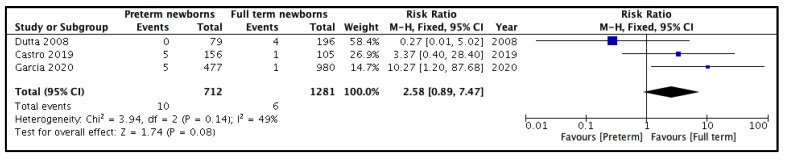
Forest plot comparison between the two patient groups in terms of the incidence of hernia recurrence.

Three out of four studies depicted this outcome [10,11,20]. The incidence of hernia recurrence was compared among 386 PT newborns (712 hernias) versus 980 FT newborns (1281 hernias). The pooled risk ratio (Figure 2) for recurrence of hernia among PT versus FT groups was 2.58 (95% CI 0.89–7.47), demonstrating no significant difference (*p* = 0.08) between the two groups. For this outcome, the heterogeneity among the included studies was neither substantial (I^2^ = 49%) nor statistically significant (*p* = 0.14).

#### 3.4.2. Meta-Analysis of Outcomes—Proportion of Children with Postoperative Complications

**Figure 3:**
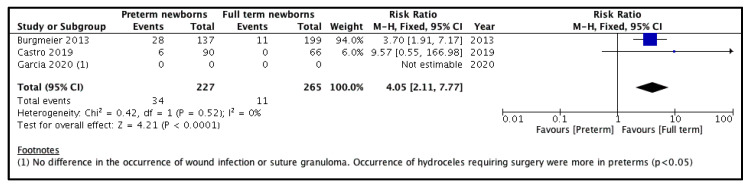
Forest plot comparison between the two patient groups in terms of the postoperative complications.

Three studies compared this outcome among the two treatment groups [9,10,11]. Of these, only two studies clearly described the number of complications [9,10]. The study by Garcia et al. [11] did not clearly mention the number of complications in both groups, however, they observed a significantly higher incidence of hydroceles requiring operative intervention in the PT group. Pooling the data from the remaining two studies (Figure 3) demonstrated a significantly higher incidence of complications in the PT group compared to the FT group (RR = 4.05, 95% CI 2.11–7.77, *p* < 0.0001). For this outcome, the estimated heterogeneity among the included studies was neither substantial (I^2^ = 0%) nor significant (*p* = 0.52).

The sub-categorization of the postoperative complications is depicted in Table 3. The PT group of newborns accounted for the majority of the major (81%) and minor (72%) events. The major complications were either *non-surgical* (i.e., severe respiratory distress requiring reintubation with prolonged ventilation (or high-frequency ventilation), seizures, bradycardia) or *surgical* (i.e., hydroceles requiring operative intervention and umbilical port-site hernia). On the other hand, the minor complications were wound infection, port-site infection, suture granuloma, and mild respiratory issues (short-term desaturation, self-limiting apnea, etc.).

## 4. Discussion

The incidence of inguinal hernia in PT newborns is closely related to the patent processus vaginalis peritonei [1,8,21]. It becomes symptomatic in cases of sliding of intra-abdominal organs into the patent processus vaginalis. The probability of developing symptomatic inguinal hernia is significantly higher in PT newborns compared to FT newborns and occurs with a frequency of 20–30% [1,2,3,21,22]. Inguinal hernia is predominantly found in male newborns, most commonly on the right side, and its frequency is inversely related to the birth weight [1,2,3,18,21,22]. A higher incidence of bilateral inguinal hernias has been found in neonates and premature newborns compared to term newborns [2,21,22,23].

Many previously published studies clearly showed that inguinal hernia represents the most common indication for surgery in children with significantly higher prevalence in PT newborns [1,2,3,4,5,8,10,19,21,22,23]. The surgical repair of inguinal hernia, even in term newborns, but especially in PT newborns, can be technically difficult because of the potential risk of a wide spectrum of complications [9,10,19,21,23,24,25]. In this specific age group, surgery is not harmless nor is anesthesia. The incidence of surgical complications in PT newborns is significantly higher, compared to FT newborns, and varies between 6–15% [23,26,27,28]. The common surgical complications in this age group include damage to spermatic cord structures, which may lead to testicular atrophy and infertility, an iatrogenic testicular ascent that may require subsequent orchiopexy, hernia recurrence, and persistent hydrocele [2,3,5,11,21,22,23,26,27,28]. A higher incidence of bleeding and wound infections has also been reported in PT compared to FT newborns [21,26,27,28].

Some authors recommend avoiding general anesthesia during the first six months of life because surgical trauma as well as inflammatory stress response may interfere with neurodevelopment [21,29]. Glial activation and neuronal hyperexcitability in cortical and subcortical areas may stimulate apoptosis and neuronal damage, especially in PT infants [21,29]. Laparoscopic inguinal hernia repair techniques are associated with a significantly lower surgical trauma and acute inflammatory stress response in comparison to open hernia repair [30]. Contrary to the above findings, some authors failed to demonstrate any significant neurodevelopmental effects of general anesthesia [31]. In addition, the incidence of cardio-respiratory complications related to tracheal intubation and general anesthesia is higher in PT infants compared to FT infants [9,25,32]. Therefore, whether general anesthesia in this specific age group is harmful or not is yet to be determined.

Considering all the above-mentioned facts, the surgical repair of inguinal hernia for PT newborns should be considered as a major procedure and performed only by consultants or senior residents under the supervision of consultants. The learning curve is longer compared to hernia surgery performed in FT infants and older children [3,6]. Generally, there is no need to rush surgery, but in PT newborns, a higher possibility of incarceration has been reported [21,27]. There is no consensus among pediatric surgeons regarding the optimal timing for inguinal hernia repair in PT newborns. Some prefer to perform surgery prior to discharge from the hospital, while others prefer to perform a delayed hernia repair [21,23]. Definitely, an early repair should take precedence over the delayed repair in cases of frequent incarcerations to avoid the risk of bowel and/or gonadal infarction and/or atrophy. A recent meta-analysis indicated no statistically significant differences in the incarceration rates, surgical, and other complications among the children who underwent an early repair versus those who underwent a delayed repair. However, the recurrence rates and respiratory difficulties were more frequent in the newborns belonging to the early repair group [33].

Next, the question of choosing the optimal surgical technique in PT newborns arises. As in FT newborns, the surgery may be performed via a conventional open or a laparoscopic approach. Most open approaches are based on the high ligation of the hernia sac. During the procedure, the damage of the vas deferens and spermatic vessels may occur while dissecting the hernia sac [3,4,22,24]. Unlike open surgery, the laparoscopic approach has several advantages and is associated with fewer complication rates. This includes the avoidance of dissecting the vas deferens and spermatic vessels, the possibility of inspection of the contralateral side, reduced pain, faster recovery, and better cosmetic outcome [3,4,5,6,7,8,10,19,20,30,34]. Several different laparoscopic techniques for pediatric inguinal hernia repair have been described, but these techniques can be divided into two major groups: traditional laparoscopic hernia repair, which includes intracorporeal suturing and requires the placement of multiple trocars, and percutaneous, needle-assisted techniques, without introducing multiple ports [5,6,10,11,35]. The choice of technique mostly depends upon the operating surgeon’s preferences and experience.

The majority of the published reports regarding the efficacy and long-term follow up of minimally invasive inguinal hernia repair in newborns are based on the data achieved in older children. Data regarding the feasibility and safety of minimally invasive surgery in PT infants and very low birth weight children are extremely limited. In the present meta-analysis, the incidence of hernia recurrence was higher among 386 PT newborns (712 hernias) compared to 980 FT newborns (1281 hernias). However, the difference could not reach statistical significance. Turial et al. [35] reported the first results of minimally invasive inguinal hernia repair in PT infants in their cohort of 58 patients. The recurrence rate was only 3.4% (2/58) in their cohort. Similar results have been demonstrated by Chan et al. [17] and Pastore et al. [19], who reported recurrence rates of 1.3% and 0%, respectively. In fact, the comparative studies by Dutta et al. [20] and Garcia et al. [11] depicted significantly higher recurrence rates in the FT group versus the PT group.

On the other hand, in this meta-analysis, a significantly higher proportion of children developed complications in the PT group versus the FT group. In fact, PT newborns accounted for the majority of the major (81%) and minor (72%) events. The major complications were either non-surgical, related to the anesthesia and tracheal intubation, or surgical (i.e., hydroceles or umbilical port-site hernia). The minor complications were mostly wound infection, port-site infection, suture granuloma, and mild respiratory issues. Similar findings have been demonstrated by previous studies. Burgmeier et al. [9] depicted a significantly higher incidence of cardiorespiratory complications in the PT versus the FT groups. Castro et al. [10] also showed significantly more events among the PT newborns. Another study comparing the complications between the PT and FT groups demonstrated a significantly higher incidence of hydroceles requiring operative intervention in the former group. In addition, Turial et al. [35] reported anesthesia-related complications in seven PT newborns. Regarding the late complications, the ascent of testes requiring subsequent orchiopexy was recorded in five cases in their study. In contrast, some studies reported no difference in the complication rates between the two groups [17,19,20]. This variation can largely be attributed to factors including the corrected age of PT newborns at surgery, size of the hernial defect, the surgeon’s experience and learning curve, etc. As there is very limited information about these factors in the included studies [9,10,11,20], well-designed randomized controlled trials are needed before any definite conclusions are drawn.

The present systematic review has few limitations. First, all the included studies had a moderate risk of bias. Second, three out of four studies had a retrospective study design. Therefore, issues related to selective reporting were evident in these studies. The main outcomes (i.e., recurrence and complication rates) were reported by three and two studies, respectively. Similarly, a non-uniform reporting of the baseline characteristics including the incidence of incarceration, the average age at surgery, etc. was also observed among the included studies. In addition, very limited information regarding the size of the hernia defect in each patient group was presented in these studies. Third, different operative techniques of laparoscopic hernia repair were utilized in these studies. Two studies utilized percutaneous needle-assisted repair [11,20] while the other two employed the intracorporeal approach for hernia repair [9,10]. Outcome differences can arise because of differences in the operative techniques. Finally, this meta-analysis involves the pooling of the data of newborns operated upon by different pediatric surgeons with different operative experience. It has been demonstrated that there exists a learning curve for laparoscopic repair of pediatric inguinal hernia. Therefore, these additional factors have to be taken into account for an optimal comparison between the two patient groups.

Despite the above limitations, the present review is the first to compare the recurrence and complication rates between the two patient groups following LHR. However, the level of evidence of the included studies is limited and prevents us from deriving an appropriate estimate of the overall effect. The strengths of the present review (as per the Downs and Black scale) include internal and external validity while the weaknesses lie in reporting and power.

## 5. Conclusions

The present meta-analysis of the published comparative studies revealed no significant difference between the PT and FT patient groups in terms of the recurrence rates following LHR. On the other hand, the incidence of postoperative complications was significantly higher among the PT newborns. However, due to moderate risk of bias among the included studies, an appropriate estimate of the overall effect could not be derived. Well-designed randomized controlled trials need to be conducted in future for an optimal comparison between the two groups.

## Figures and Tables

**Table 1:** Baseline characteristics of the included studies.

Author	Study Design	No of Patients PT FT		No of Hernias PT FT		% of B/L Hernias PT FT		Incarceration Rate PT FT		Average Gestational Age (Weeks) or Postnatal Age (Days) at Surgery PT FT	
Dutta et al., 2008 [19]	Pro	49	138	79	196	25 children with B/L hernia *		18%	4%	*	
Burgmeier et al., 2013 [9]	Retro	137	199	*	*	*		*		69 **	45 **
Castro et al., 2019 [10]	Retro	90	66	156	105	58%	42%	26%	24%	63 (0–365) ^§^	139 (16–351) ^§^
Garcia et al., 2020 [11]	Retro	247	776	477	980	61%	27%	*		45.7 ^†^ (28–59)	3.5 (+/−0.1) years

* Data according to subgroups not mentioned; ** Average postnatal age (in days). Range/SD not mentioned. ^§^ Mean (range); in days; ^†^ Mean (range) gestational age in weeks. Pro—prospective cohort, Retro—retrospective study, PT—preterm newborns, FT—full term newborns, B/L—bilateral.

**Table 2:** Downs and Black scale scores for the included studies by author 1 and author 2. The total scores and inter-observer agreement are also depicted in the table.

Methodological Assessment by Author 1						
Study	Reporting	External Validity	Internal Validity-Bias	Internal Validity-Confounding	Power	Total Scores
Dutta et al., 2008 [20]	6	3	4	6	0	16
Burgmeier et al., 2013 [9]	7	3	5	6	0	18
Castro et al., 2019 [10]	8	3	5	6	0	19
Garcia et al., 2020 [11]	10	3	5	6	0	21
**Methodological Assessment by Author 2**						
**Study**	**Reporting**	**External Validity**	**Internal Validity-Bias**	**Internal Validity-Confounding**	**Power**	**Total Scores**
Dutta et al., 2008 [20]	6	3	4	6	0	16
Burgmeier et al., 2013 [9]	7	3	5	6	0	18
Castro et al., 2019 [10]	9	3	5	6	0	20
Garcia et al., 2020 [11]	10	3	5	6	0	21
**Total Scores and Inter-OBSERVER Agreement**						
**Study**	**Rater 1**	**Rater 2**	**Mean**	**Kappa Value**	***p*-Value**	
Dutta et al., 2008 [20]	16	16	16	0.978	<0.001	
Burgmeier et al., 2013 [9]	18	18	18			
Castro et al., 2019 [10]	19	20	19.5			
Garcia et al., 2020 [11]	21	21	21			

**Table 3:** Postoperative complications among the preterm and full-term infants.

Study	Preterm Newborns (*n*)		Full-Term Newborns (*n*)	
	Major ^§^	Minor ^§^	Major ^§^	Minor ^§^
Dutta 2008 * [20]	-	-	-	-
Burgmeier 2013 [9]	10	18	3	8
Castro 2019 [10]	3	3	0	0
Garcia 2020 ^#^ [11]	^7 †^	-	24 ^†^	-

^§^ The complications were originally divided on the basis of the Clavien–Dindo classification. For an easy interpretation, these were further grouped into major (Grade 3 or above) and minor (Grade 1 and 2); * Subgroup comparison of the complications was not mentioned; ^#^ A clear description of the major and minor complications was not available; ^†^ A comparison of the total complications requiring operative intervention showed no difference. Although the incidence of wound infection and suture granuloma were similar, the hydroceles requiring operative intervention were higher in the preterm group (*p* < 0.05).

## Data Availability

The data presented in this study are available upon request of the respective author.

## Appendix A

PubMed—(“laparoscopes”[MeSH Terms] OR “laparoscopes”[All Fields] OR “laparoscope”[All Fields] OR “laparoscopical”[All Fields] OR “laparoscopically”[All Fields] OR “laparoscopics”[All Fields] OR “laparoscopy”[MeSH Terms] OR “laparoscopy”[All Fields] OR “laparoscopic”[All Fields]) AND (“herniorrhaphy”[MeSH Terms] OR “herniorrhaphy”[All Fields] OR (“hernia”[All Fields] AND “repair”[All Fields]) OR “hernia repair”[All Fields]) AND (“premature birth”[MeSH Terms] OR (“premature”[All Fields] AND “birth”[All Fields]) OR “premature birth”[All Fields] OR “preterm”[All Fields] OR “preterms”[All Fields])

EMBASE—(‘laparoscopic hernia repair’:ti,ab,kw) AND (‘preterm’:ti,ab,kw)

SCOPUS—TITLE-ABS-KEY(‘laparoscopic hernia repair’) AND TITLE-ABS-KEY(‘preterm’)

Web of Science—(ALL = (laparoscopic hernia repair)) AND ALL = (preterm)

**Table A1:** Results of the search strategy.

Database	Studies
PubMed	21
EMBASE	4
SCOPUS	49
Web of Science	15
Total	89
Duplications	28
After duplication removal	61

## References

[1] Burgmeier C., Dreyhaupt J., Schier F. (2014). Comparison of inguinal hernia and asymptomatic patent processus vaginalis in term and preterm infants. J. Pediatr. Surg..

[2] Ein S.H., Njere I., Ein A. (2006). Six thousand three hundred sixty-one pediatric inguinal hernias: A 35-year review. J. Pediatr. Surg..

[3] Pogorelić Z., Rikalo M., Jukić M., Katić J., Jurić I., Furlan D., Budimir D., Biočić M. (2016). Modified Marcy repair for indirect inguinal hernia in children: A 24-year single-center experience of 6826 pediatric patients. Surg. Today.

[4] Kantor N., Travis N., Wayne C., Nasr A. (2019). Laparoscopic versus open inguinal hernia repair in children: Which is the true gold-standard? A systematic review and meta-analysis. Pediatr. Surg. Int..

[5] Pogorelić Z., Čohadžić T., Jukić M., Biliškov A.N. (2020). Percutaneous Internal Ring Suturing for the Minimal Invasive Treatment of Pediatric Inguinal Hernia: A 5-Year Single Surgeon Experience. Surg. Laparosc. Endosc. Percutan. Tech..

[6] Pogorelić Z., Huskić D., Čohadžić T., Jukić M., Šušnjar T. (2021). Learning Curve for Laparoscopic Repair of Pediatric Inguinal Hernia Using Percutaneous Internal Ring Suturing. Children.

[7] Parelkar S.V., Oak S., Gupta R., Sanghvi B., Shimoga P.H., Kaltari D., Prakash A., Shekhar R., Gupta A., Bachani M. (2010). Laparoscopic inguinal hernia repair in the pediatric age group—experience with 437 children. J. Pediatr. Surg..

[8] De Goede B., Verhelst J., van Kempen B.J., Baartmans M.G., Langeveld H.R., Halm J.A., Kazemier G., Lange J.F., Wijnen R.M. (2015). Very low birth weight is an independent risk factor for emergency surgery in premature infants with inguinal hernia. J. Am. Coll. Surg..

[9] Burgmeier C., Schier F. (2013). Cardiorespiratory complications after laparoscopic hernia repair in term and preterm babies. J. Pediatr. Surg..

[10] Aneiros Castro B., Cano Novillo I., Garcia Vazquez A., de Miguel Moya M. (2019). Is the laparoscopic approach safe for inguinal hernia repair in preterms?. J. Laparoendosc. Adv. Surg. Tech. A.

[11] Garcia D.I., Baker C., Patel S., Hebra A.V., Cina R.A., Streck C.J., Lesher A.P. (2021). Long-term outcomes of pediatric laparoscopic nee-dled-assisted inguinal hernia repair: A 10-year experience. J. Pediatr. Surg..

[12] Moher D., Liberati A., Tetzlaff J., Altman D.G. (2009). For the PRISMA Group Preferred reporting items for systematic reviews and meta-analyses: The PRISMA statement. BMJ.

[13] Clavien P.A., Barkun J., de Oliveira M.L., Vauthey J.N., Dindo D., Schulick R.D., de Santibañes E., Pekolj J., Slankamenac K., Bassi C. (2009). The Clavien-Dindo classification of surgical complications: Five-year experience. Ann. Surg..

[14] Downs S.H., Black N. (1998). The feasibility of creating a checklist for the assessment of the methodological quality both of randomised and non-randomised studies of health care interventions. J. Epidemiol. Community Health.

[15] Landis J.R., Koch G.G. (1977). The measurement of observer agreement for categorical data. Biometrics.

[16] (2021). Cochrane Handbook for Systematic Reviews of Interventions (version 6.2). Cochrane. www.training.cochrane.org/handbook.

[17] Chan I.H., Lau C.T., Chung P.H., Chan K.L., Lan L.C., Wong K.K., Tam P.K. (2013). Laparoscopic inguinal hernia repair in premature neonates: Is it safe?. Pediatr. Surg. Int..

[18] Steven M., Greene O., Nelson A., Brindley N. (2010). Contralateral inguinal exploration in premature neonates: Is it necessary?. Pediatr. Surg. Int..

[19] Pastore V., Bartoli F. (2014). Neonatal laparoscopic inguinal hernia repair: A 3-year experience. Hernia.

[20] Dutta S., Albanese C. (2008). Transcutaneous laparoscopic hernia repair in children: A prospective review of 275 hernia repairs with minimum 2-year follow-up. Surg. Endosc..

[21] Prato A.P., Casaccia G., Arnoldi R. (2017). Timing and management of inguinal hernia in the premature baby. Eur. J. Pediatr. Surg..

[22] Crankson S.J., Al Tawil K., Al Namshan M., Baylon B.J., Gieballa M., Al Jadaan S., Ahmed I.H. (2015). Management of inguinal hernia in premature infants: 10-year experience. J. Indian Assoc. Pediatr. Surg..

[23] Wang K.S. (2012). Committee on Fetus and Newborn, American Academy of Pediatrics, Section on Surgery, American Academy of Pediatrics. Assessment and management of inguinal hernia in infants. Pediatrics.

[24] Liu G., Zhang W., Zhou J., Sun B., Jiang B., Wang H. (2020). Laparoscopic versus open herniorrhaphy for children with inguinal hernia: A meta-analysis of randomized controlled trials. Medicine.

[25] Nevešćanin A., Vickov J., Elezović Baloević S., Pogorelić Z. (2020). Laryngeal mask airway versus tracheal intubation for laparoscopic hernia repair in children: Analysis of respiratory complications. J. Laparoendosc Adv. Surg. Tech. A.

[26] Takahashi A., Toki F., Yamamoto H., Otake S., Oki Y., Kuwano H. (2012). Outcomes of herniotomy in premature infants: Recent 10 year experience. Pediatr. Int..

[27] Hughes K., Horwood J.F., Clements C., Leyland D., Corbett H.J. (2016). Complications of inguinal herniotomy are comparable in term and premature infants. Hernia.

[28] Rosenberg J. (2007). Pediatric inguinal hernia repair—A critical appraisal. Hernia.

[29] Sanders R.D., Hassell J., Davidson A.J., Robertson N.J., Ma D. (2013). Impact of anaesthetics and surgery on neurodevelopment: An update. Br. J. Anaesth..

[30] Jukić M., Pogorelić Z., Šupe-Domić D., Jerončić A. (2018). Comparison of inflammatory stress response between laparoscopic and open approach for pediatric inguinal hernia repair in children. Surg. Endosc..

[31] Davidson A.J., Disma N., de Graaff J.C., Withington D.E., Dorris L., Bell G., Stargatt R., Bellinger D.C., Schuster T., Arnup S.J. (2016). GAS consortium. Neurodevelopmental outcome at 2 years of age after general an-aesthesia and awake-regional anaesthesia in infancy (GAS): An international multicentre, randomised controlled trial. Lancet.

[32] Biliškov A., Ivančev B., Pogorelić Z. (2021). Effects on Recovery of Pediatric Patients Undergoing Total Intravenous Anesthesia with Propofol versus Ketofol for Short—Lasting Laparoscopic Procedures. Children.

[33] Masoudian P., Sullivan K.J., Mohamed H., Nasr A. (2019). Optimal timing for inguinal hernia repair in premature infants: A systematic review and meta-analysis. J. Pediatr. Surg..

[34] Shibuya S., Fujiwara N., Ochi T., Wada M., Takahashi T., Lee K.D., Miyazaki E. (2019). The learning curve of laparoscopic percutaneous extraperitoneal closure (LPEC) for inguinal hernia: Protocoled training in a single center for six pediatric surgical trainees. BMC Surg..

[35] Turial S., Enders J., Krause K., Schier F. (2010). Laparoscopic inguinal herniorrhaphy in premature infants. Eur. J. Pediatr. Surg..

